# Molecular and Functional Characterization of Odorant-Binding Protein Genes in an Invasive Vector Mosquito, *Aedes albopictus*

**DOI:** 10.1371/journal.pone.0068836

**Published:** 2013-07-23

**Authors:** Yuhua Deng, Hui Yan, Jinbao Gu, Jiabao Xu, Kun Wu, Zhijian Tu, Anthony A. James, Xiaoguang Chen

**Affiliations:** 1 Key Laboratory of Prevention and Control of Emerging Infectious Diseases of Guangdong Higher Education Institutes, School of Public Health and Tropical Medicine, Southern Medical University, Guangdong, PR China; 2 Department of Biochemistry, Virginia Tech, Blacksburg, Virginia, United States of America; 3 Departments of Microbiology & Molecular Genetics and Molecular Biology & Biochemistry, University of California Irvine, Irvine, California, United States of America; Goethe University Frankfurt, Germany

## Abstract

*Aedes albopictus* is a major vector of dengue and Chikungunya viruses. Olfaction plays a vital role in guiding mosquito behaviors and contributes to their ability to transmit pathogens. Odorant-binding proteins (OBPs) are abundant in insect olfactory tissues and involved in the first step of odorant reception. While comprehensive descriptions are available of OBPs from *Aedes aegypti*, *Culex quinquefasciatus* and *Anopheles gambiae*, only a few genes from *Ae. albopictus* have been reported. In this study, twenty-one putative AalbOBP genes were cloned using their homologues in *Ae. aegypti* to query an *Ae. albopictus* partial genome sequence. Two antenna-specific OBPs, AalbOBP37 and AalbOBP39, display a remarkable similarity in their overall folding and binding pockets, according to molecular modeling. Binding affinity assays indicated that AalbOBP37 and AalbOBP39 had overlapping ligand affinities and are affected in different pH condition. Electroantennagrams (EAG) and behavioral tests show that these two genes were involved in olfactory reception. An improved understanding of the *Ae. albopictus* OBPs is expected to contribute to the development of more efficient and environmentally-friendly mosquito control strategies.

## Introduction

Dengue is an emerging acute infectious disease transmitted by *Aedes* mosquitoes and is a serious global public health problem [1]. The principal dengue vector species, *Ae. aegypti* and *Ae. albopictus*, share a number of genome features [2]. These two species differ in large-scale geographical distribution, but share breeding-site preferences, mating behavior, relative seasonal abundance and high anthropophilic and endophilic feeding behavior.

While extensive research has been conducted on the behavior, ecology and genomics of *Ae. aegypti*, *Cx. quinquefasciatus* and *An. gambiae*, relatively less is knew about *Ae. albopictus*, especially the olfactory system. Olfaction plays a crucial role in guiding insect behaviors such as host-seeking and oviposition, and odorant receptors (ORs) and odorant-binding proteins (OBPs) have key roles in these processes [3]–[5]. OBPs are involved in the earliest steps of odorant reception during which they bind, solubilize and deliver odorant molecules to ORs [6], [7]. The first insect OBP was discovered in the giant moth, *Antheraea polyphemus* [5] and the first mosquito OBP, CquiOBP1, was isolated from antennae of female *Cx. quinquefasciatus* [8]. To date, 33, 34 and 55 classic OBPs have been identified in *An. gambiae*, *Ae. aegypti* and *Cx. quinquefasciatus*, respectively [9]–[12]. These OBPs share characteristic features, such as small size and presence of an N-terminal signal peptide, as well as the highly conserved pattern of six cysteine residues called the “classic motif” [11], [12]. However, few *Ae. albopictus* OBPs have been reported and these have not been clustered as classic OBPs [13], [14].

Numerous physiological activities and ligands for insect OBPs have been identified. CquiOBP1 is a mosquito oviposition-sensitive (MOP) OBP [15]. RNA interference (RNAi)-mediated knock-down of CquiOBP1 produces mosquitoes with reduced reception to oviposition attractants. Knock-down experiment with the AgamOBP1 shows that mosquito responses to indole are impaired [16], [17]. LUSH odorant-binding protein mediates chemosensory responses to alcohols in *Drosophila melanogaster* and mutants lose the capacity to avoid concentrated ethanol. All of these studies support a critical role for OBPs in the insect olfactory system [18].

Twenty-one putative OBP genes were cloned and identified from *Ae. albopictus* in the research described here. Further functional characterizations of two antenna-specific OBPs, AalbOBP37 and AalbOBP39, showed that they were involved significantly in olfactory reception. The studies of *Ae. albopictus* OBPs are expected to support a deeper understanding of the molecular basis of olfaction in this species.

## Materials and Methods

### Ethics Statement

All vertebrate animals were housed and handled in strict accordance with the guidelines of the institutional and national Committees of Animal Use and Protection. All experimental procedures on mice were approved by the Committee on the Ethics of Animal Experiment of Southern Medical University (Permit Number: SCXK 2006-0015).

### Mosquitoes

The *Ae. albopictus* strain used in this study was isolated in 1981 from the wild in Foshan County, Guangdong Province, which was obtained from the CDC of Guangdong Province (China). Mosquitoes were reared at 27°C with 70–80% relative humidity and a 16 h: 8 h photoperiod. Larvae were fed on yeast powder while adults were maintained on a 10% sugar solution. Adult female mosquitoes were fed on blood of anesthetized mouse which was then kept back to the animal room.

### Molecular Cloning of AalbOBPs

*Aedes aegypti* (AaegOBP) protein sequences were used as a query to blast a partial genome sequence of *Ae. albopictus* using TBLASTN [19], [20]. The resulting partial sequences were used to design gene-specific primers (GSPs) for 5′- or 3′-end RACE (Table S1 and Table S2, respectively). Total RNA was prepared from 5∼7 days-old adults using the TRIzol Reagent (Invitrogen, Carlsbad, CA) and made into cDNA using a SMART RACE cDNA amplification kit (Clontech, Mountain View, CA) and the 5′-Full RACE Core Set (Takara, Japan). PCR reactions were performed using universal primer (Table S1 and Table S2) and GSPs, and the products were cloned into the pMD-18T vector (Takara, Japan) and sequenced. The putative AalbOBP cDNA sequences have been deposited into GenBank under accession numbers provided in Table 1.

**Table 1:** Structural characteristics of putative AalbOBPs.

OBP name	GenBank Accession	Amino-acid	MW	Cysteine spacing	Signal peptide(%)	CDD prediction(E-value)
**AalbOBP5**	KC405539	260/243	28.415	43/3/43/20/9/8/9	100	NO CDD
AalbOBP10	KC405540	140/115	13.127	28/3/38/7/8	99.9	PBP_GOBP(3e-18)
AalbOBP11	KC405541	137/119	13.089	26/3/38/7/8	99.9	PBP_GOBP(4e-20)
AalbOBP13	KC405542	133/115	12.529	27/3/38/8/8	100	PBP_GOBP(1e-20)
AalbOBP14	KC405543	133/115	12.529	27/3/38/8/8	100	PBP_GOBP(5e-26)
AalbOBP19	KC405544	145/119	13.784	26/3/38/10/8	99.4	PBP_GOBP(6e-12)
AalbOBP20	KC405545	166/142	15.922	29/3/33/8/8	99.7	PBP_GOBP(1.2e-12)
AalbOBP21	KC405546	146/128	14.784	26/3/40/12/8	98.7	PBP_GOBP(5.4e-20)
**AalbOBP24**	KC405547	200/180	20.896	43/3/43/17/9/8/9	100	NO CDD
AalbOBP25	KC405548	200/180	20.941	43/3/43/17/9/8/9	100	NO CDD
AalbOBP37	KC405549	149/131	15.281	27/3/38/8/8	100	PBP_GOBP(3.1e-16)
AalbOBP38	KC405550	140/124	14.566	26/3/37/8/8	99.9	PBP_GOBP(2.8e-22)
AalbOBP39	KC405551	143/125	14.604	26/3/37/8/8	99.7	PBP_GOBP(1.3e-24)
**AalbOBP42**	KC405552	157	17.892	36/3/42/26/9/8/9	NO	NO CDD
AalbOBP43	KC405553	193/173	19.508	36/3/42/26/9/8/9	99.3	NO CDD
AalbOBP55	KC405554	151/130	14.249	26/3/40/10/8	91.9	PBP_GOBP(2.0e-21)
AalbOBP56	KC405555	143/125	14.591	26/3/37/8/8	99.8	PBP_GOBP(7.2e-20)
AalbOBP59	KC405556	166/142	15.921	29/3/33/8/8	99.7	PBP_GOBP(5.1e-13)
AalbOBP61	KC405557	133/115	12.529	27/3/38/8/8	100	PBP_GOBP(5.2e-26)
**AalbOBP62**	KC405558	193/173	19.402	36/3/42/26/9/8/9	99.3	NO CDD
**AalbOBP63**	KC405559	157	17.892	36/3/42/26/9/8/9	NO	NO CDD

The number of amino acids is indicated for complete/mature proteins. The Plus-C OBPs are colored in bold.

### Bioinformatics Analysis of Mosquito OBPs

Signal peptides present in the full-length conceptual translation products were predicted with the SignalP4.0 Server. The calculated molecular weights (MW) and isoelectric points (IP) were obtained using the ExPASy proteomics server [21] and PBP_GOPB motifs were identified using NCBI conserved domains database (CDD). The amino acid sequences of the 21 putative AalbOBP were aligned to themselves using clustalW2 [21] and also to the 33, 34 and 55 classic OBPs identified in *An. gambiae* [9], [10], *Ae. aegypti* [11], [22] and *Culex quinquefasciatus* [12], respectively. The amino acid sequences were used to create an entry file for phylogenetic analysis in MEGA 4.0.2 [12]. An un-rooted consensus neighbor-joining tree was calculated at default settings with pair-wise gaps deletions [23].

### Expression Profiles and Molecular Modeling of AalbOBPs

*Aedes albopictus* tissues, such as antennae, maxillary palps, proboscises, legs and bodies (tissues remaining after organ removal), were dissected from 5∼7 day-old adult females. Total RNA was isolated as described above and converted into cDNA using Prime Script^R^ RTase (Takara, Japan). The integrity of each cDNA sample was confirmed by amplification of fragments corresponding to the *Ae. albopictus* β-actin gene (DQ657949). GSPs of putative AalbOBPs were designed (Table S3) and PCR using equivalent amounts of cDNA template was carried out in a T-Gradient Thermoblock (Applied Biometra, German). Amplification products were resolved in ethidium bromide-stained agarose gels and visualized using a Gel DOC XR Molecular Imager (BioRad, USA).

Three-dimensional models of OBPs were generated using the on-line program SWISS MODEL [24], [25]. The structure of CquiOBP1 bound with the MOP pheromone (PDB: 2L2C_A) was used as a template for AalbOBP37. Amino acid identity between the two proteins is 34%. The template for AalbOBP39 was *Ae. aegypti* OBP1 (accession number 3K1E_B; 98% identity between the two proteins). Models were displayed using the SwissPdb Viewer programme “Deep-View” [26], [27].

### Construction of Expression Vectors and Preparation of Recombinant Proteins

Forward and reverse GSPs containing enzyme sites (*Msc*I and *Hind*III, respectively) were designed based on open reading frames of AalbOBP37 and AalbOBP39 (Table S4-A). PCR was performed using *Pfu* DNA polymerase (Tiangen, China) and the products were purified, ligated into pMD-18T (Takara, Japan) and sequenced. The plasmid vector pET-22b(+) was used for periplasmic expression [28] of recombinant proteins containing a 6xHis-tag fusion [17]. pMD-18T containing the appropriate open reading frame inserts and pET-22b(+) were digested with *Msc*I and *Hind*III (Fermentas, USA) at 37°C for 15 min. After purification using Gel Extraction Kit (OMEGA, USA), the target fragments were ligated into pET-22b(+) with T4 DNA ligase (Takara, Japan). The resulting plasmids were sequenced and shown to encode the mature proteins.

BL21 (DE3) cells transformed with plasmids encoding the recombinant protein were induced by IPTG in LB medium at 37°C. Cells were harvested 4 hours post-induction and sonicated. Soluble proteins were purified over a Ni-NTA Superflow Cartridge (QIAGEN, Valencia, CA), followed by gel-filtration (GE-Healthcare) [29], [30]. Purified proteins were concentrated using a Centriprep YM-3 (Millipore, USA). The concentration of the recombinant protein was measured and the integrity of the final protein samples confirmed by SDS-PAGE and western blot using his-tag antibody (data not show).

### Fluorescence Binding Assays

Four known ligands and a reporter ligand, N-phenyl-1-naphthylamine (1-NPN), were used in fluorescence binding assays to investigate binding affinities of AalbOBP37 and AalbOBP39. Affinities for 1-NPN were measured first utilizing 25 ug/ml protein solutions prepared in 20 mM sodium acetate, pH 7.4. Increasing doses of 1-NPN (3.2 mM in methanol [31], [32]) were added to the protein solutions until the fluorescence intensity saturated. The amount of 1-NPN added was recorded and the fluorescence intensity was used as a reference (100%) to normalize subsequent experiments. Based on 25 ug/ml AalbOBP37 at pH 7.4, 20 ul of 1-NPN was added to reach fluorescence intensity saturation (final concentration of 1-NPN: 32 uM), and 19 ul of 1-NPN was added for 25 ug/ml AalbOBP39 saturation (final concentration of 1-NPN: 30.4 uM) (Fig. S1). To detect whether the binding affinities of target proteins to selected ligands (3.2 mM in methanol) exhibiting dose-dependent manner, we just selected four different final concentrations (0 uM, 6.4 uM, 12.8 uM, 19.2 uM) and the fluorescence intensities were recorded and normalized to the 1-NPN reference.

To abolish the non-specific results, we selected the protein of BL21 (DE3) cells as negative control to perform binding affinity assay. The results indicated that only target OBPs could bind 1-NPN, which showed increasing fluorescent intensity, comparing with negative control (Fig. S1B). The proteins of *E.coli* showed no binding affinity to 1-NPN. The fluorescent of *E.coli* protein may derive from the intrinsic fluorescence of tryptophan residues (Fig. S1B) [33].

Fluorescence measurements were performed on a spectrofluorophotometer (RF-5301, Shimadzu, Kyoto, Japan) at 25±1°C in a right angle configuration with a 1 cm light path quartz cuvette. Samples in 2-ml cell were excited at 337 nm and emission spectra were recorded from 350–600 nm, with emission and excitation slit widths of 3 nm and 10 nm, respectively.

### RNAi and qRT-PCR

GSPs containing T7 promoter sequences were designed (Table S4-B) and dsRNA was synthesized at 37°C for 16 hours using the Ambion MEGAscript RNAi Kit (Ambion, USA). The dsRNA products were purified, their integrity confirmed by gel electrophoresis, and the concentration was determined by a Smart Spec^TM^ 3000 NanoPhotometer (Bio-Rad, USA).

The dsRNA of target genes (∼800–900 ng) was injected through the intersegmental thoracic membrane of 24 h-old adult females with a drawn out capillary (1 mm o.d.) with a 40 mm tip aperture connected via Teflon tubing to a 50 ml syringe mounted to a syringe pump. Total RNA was extracted from individual mosquitoes at 4 days post-injection. Residual DNA was removed with RNase-free DNaseI treatment and cDNA synthesized as described above. In RNAi experiment, the *Ae. albopictus* β-actin gene was used as the reference for qRT-PCR. The choice of this reference gene was based on previous reports on quantitative analysis of the expression of *Ae. albopictus* genes, including OBP genes [14], [34]. GSPs for qRT-PCR were designed using the Beacon Designer software 7.5 (Primer Biosoft International, Palo Alto, CA, USA) (Table S4-C).

Triplicate quantitative gene amplification reactions were performed using miScript SYBR^R^ Green PCR Kit (QIAGEN, Valencia, CA) and the products run on a MX3005P^TM^ Real Time PCR System (Stratagene, La Jolla, CA, USA). The qRT-PCR results were analyzed using the 2^−ΔΔCT^ method as described [35]. The amplification conditions were denaturation at 95°C for 15 min, followed by 40 cycles of 95°C for 15 s, 62°C for 30 s, and 72°C for 30 s.

### Electrophysiological Recording

Antennae were excised from the heads of 4–5 days-old females and placed on a reference electrode coated with electrode gel (Spectra 360, Parker Laboratories, INC, USA). The distal end of the antennae, cut to ensure a good electrical contact, was placed carefully on the recording electrode (Syntech, Germany). Electroantennagram (EAG) signals were run through a 10×amplifier and processed with a PC-based interface and software package (Syntech, Germany).

We selected four previously reported compounds, 1-octen-3-ol, indole, 3-methy indole (skatole) and DEET to perform EAG analysis. All chemicals were purchased from J&K Company (Beijing, China) and were ≥98% pure. The compounds were mixed with distilled hexane using a double-dilution method to a final concentration of 2.5–10 ug/ul. A 10 ul aliquot of each solution was applied to a filter paper strip and the solvent evaporated before the paper strip was inserted into a Pasteur pipette. A 500 ms pulse (5 ml/s) from a stimulus controller, CS-55 (Syntech, Germany), was used to deliver the chemical stimulants to a continuous humidified airflow (10 ml/s) over the EAG preparation. The chemicals were tested randomly and applied with 1 min interval between stimulations. Initial screening was performed using 25 ug, 50 ug and 100 ug source dose solution. Hexane was used as control. Six to eight mosquitoes were tested for each compound to calculate the average EAG amplitude and standard deviation.

1-octen-3-ol is a host-derived kairomone identified from human sweat [36] that elicits a significant EAG response from female mosquito antennae [21], [36]. To assess the relative response of the other three compounds, their EAG responses were normalized using 1-octen-3-ol as reference (100%). The 100 ug source dose solutions of these three compounds elicited up to 50% EAG response compared to 1-octen-3-ol and were selected for further analysis to investigate the effect of AalbOBP37 and AalbOBP39 knockdown on the EAG response. Antennae for EAG detection were excised from the heads of female mosquitoes 4–5 days after injecting dsRNAs against either AalbOBP37 or AalbOBP39 [15], [17]. EAG mean response was shown from six to eight individual mosquitoes. Non-injected mosquitoes were used as normal control, and mosquitoes injected with dsRNA for dsRED were used as negative control.

### Behavior Experiments

Flight orientation behavior of mosquitoes was observed in a Y-tube olfactometer with stem and arms of 100 and 63 centimeters in length, respectively. A methodology derived from previous research [37] was followed with some modifications. Each compound tested was one of the EAG-active ligands described in our study.

Thirty female mosquitoes were transferred as a group into the releasing chamber and kept for a moment for acclimatization. A filter paper applied with a 10 ul aliquot of each compound (compound dose: 25 ug) was loaded onto the odor cartridge (treatment chamber). The control arm housed a cartridge with solvent only. Persistent airflow introduced into both arms carried the odor of the tested compound as well as control stimulus downwind into the stem region. The orientation behavior and number of mosquitoes caught or exhibiting flight activity toward either odor plume flowing from the respective arms were observed after 10 minutes. Mosquitoes remaining at the stem of the Y-tube olfactometer were not considered in the analysis of repellency.

The repellency of each compound was estimated by the repellency index (R). This index was calculated using the formula R = (C-T)/(C+T), where T indicates the number of mosquitoes in the treatment arm and C those in the control arm [31]. Therefore, a value of R = 1 or -1 indicates that all the insects are found in the control or treatment arm, respectively. R = 0 corresponds to a situation where the mosquitoes distributed equally between two arms and indicates no effect of the tested substance.

The position of stimulus and solvent was exchanged between each replicate to avoid an orientation effect of the mosquitoes. Six to eight replications with each compound were performed at room temperature (27–30°C). The olfactometer was washed with water between every replicate and dried for 2–3 hours to remove odors of previous trials.

Chemotaxis of 30 mosquitoes to indole was performed using dsRNA-injected and non-injected mosquitoes. The method was described above, except for a minor difference. Both olfactometer’s arm housed the cartridge with an equivalent quantity of indole (12.5 ug each arm). The number of mosquitoes caught or exhibiting flight activity toward persistent odor plume in both arms was considered in the analysis after 10 min. Data were calculated from six to eight repetitions. Non-injected mosquitoes were used as normal control (100% reference), and mosquitoes injected with dsRNA for dsRED were used as negative control.

### Statistical Analysis

The qRT-PCR results of post-RNAi and the chemiotaxis activity of mosquitoes in the behavior experiment were assessed by a one-way analysis of variance (ANOVA) followed by the Fish’s least significant difference test (LSD) procedure (homogeneity of variance: P>0.05). EAG responses in the control and knock-down mosquitoes were evaluated by one-way ANOVA followed by the Games-Howell procedure (homogeneity of variance: P<0.05). The repellency of each compound tested was analysed on the basis of total number of mosquitoes in each olfactometer’s arm by the chi-square goodness-of-fit test. The test assesses whether a significant difference exists between the observed number of mosquitoes in each arm of the olfactometer and the expected number based upon the null hypothesis of a 50∶50 mosquito’s distribution. Asterisks indicate the statistical significance: *P<0.05, **P<0.01, ***P<0.001. Error bars show standard deviation. The SPSS computer software version 13.0 (SPSS Inc., Chicago, IL) was used for data analysis.

## Results and Discussion

### Cloning, Molecular Characterization and Classification of Putative AalbOBPs

**Figure 1:**
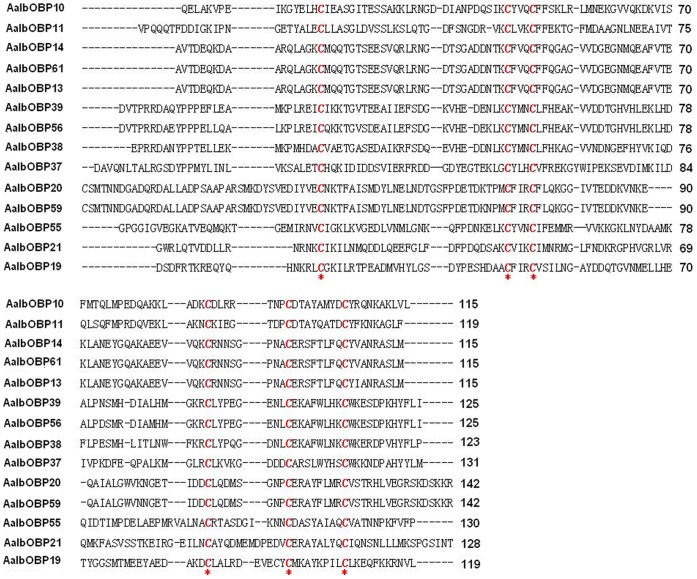
Amino acid alignment of *Ae. albopictus* Classic OBPs. Mature amino acids sequences displayed the high overall divergence of AalbOBPs family as only the six cysteine residues were completely conserved among all proteins. Conserved cysteine residues were highlighted in red and asterisk.

Twenty-one putative full-length AalbOBP genes (according to the nomenclature of their homologous gene, designated AalbOBP5, 10, 11, 13, 14, 19, 20, 21, 24, 25, 37, 38, 39, 42, 43, 55, 56, 59, 61, 62 and 63), containing 5′- and 3′-untranslated regions, were cloned successfully using the partial genomic sequences of *Ae. albopictus* and *Ae. aegypti* OBP sequences. AalbOBP conceptual translation products have high identity in amino acid sequence with their respective *Ae. aegypti* homologues (Table 1). The putative AalbOBPs share the characteristic features of known mosquito OBPs including signal peptides, cysteine spacing, molecular weights and CDD predictions [11]–[13], [21], [38]. These characteristics support classifying 21 genes as encoding OBPs, and their nomenclature follows the homologous genes in *Ae. aegypti* [11], [13]. The mature amino acids sequences of the cloned AalbOBPs displayed high overall divergence amongst themselves with only the six cysteine residues characteristic of classic OBPs conserved among all proteins (Fig. 1).

**Figure 2:**
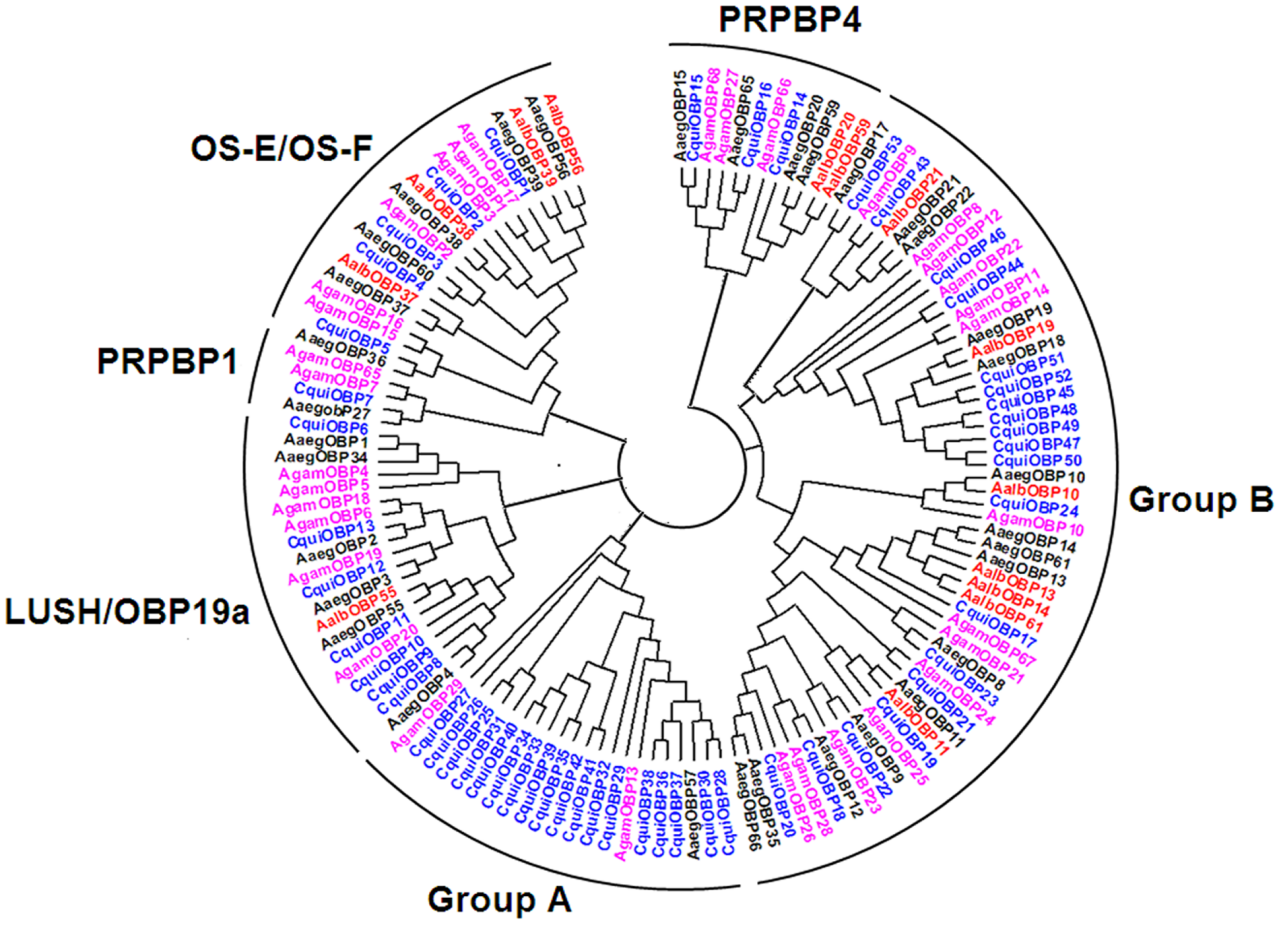
Phylogenic analysis of mosquito classic OBP amino acid sequences. 33 classic AgamOBPs (pink), 53 classic CquiOBPs (blue), 34 classic AaegOBPs (black), and 14 putative AalbOBPs (red) were grouped into OS-E/OS-F, PBPRP1, PBPRP4, LUSH/OBP19a, Group A and Group B. AalbOBP37 and AalbOBP39 were grouped into OS-E/OS-F.

A consensus sequence comparison tree was constructed by the neighbor joining method with 1000 bootstrap replicates [23] using the 33, 34 and 53 classic OBPs identified from *An. gambiae, Ae. aegypti* and *C*x. *quinquefasciatus*, respectively. Fourteen of the twenty-one OBPs were clustered into six different groups of classic OBPs (Fig. 2), that is a small molecular protein containing signal peptide and six conservative cysteine residues. AalbOBP37 and AalbOBP39 were assigned to the OS-E/OS-F group, which contains proteins knew to be olfactory-specific OBPs [17], [28], [39]. Additionally, CDD predictions also showed that these fourteen proteins cluster with members of the PBP_GOBP family (Table 1). The remaining seven genes were identified as Plus-C OBP based on their motifs [11], [38], [40] (Table S5, Fig. S2). It is likely that more OBPs are present in *Ae. albopictus* and the complete repertoire awaits discovery when the genome project is finished.

### Expression Profiles of AalbOBP Genes

**Figure 3:**
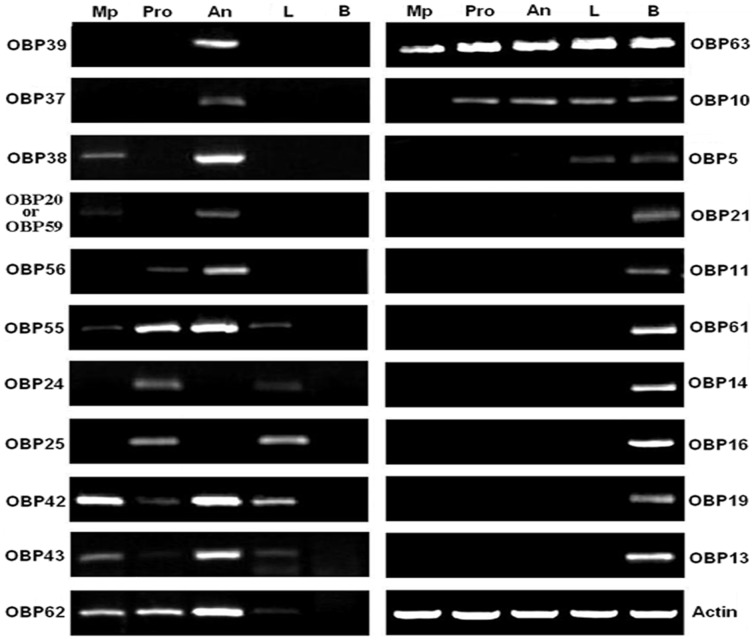
RT-PCR analysis of *Ae. albopicus* OBPs in different tissues. AalbOBP37 and AalbOBP39 were exclusively detected in the antennae, while others were found in olfactory and/or non-olfactory tissues. Because of high identity of nucleotide between AalbOBP20 and AalbOBP59 (96.8%), they were showed together. Mp, maxillary palps; Pro, proboscises; An, antennae; L, Legs and B, bodies.

A number of OBP-like proteins expressed in non-olfactory tissues are associated with olfactory functions. We grouped antennae, maxillary palps and proboscises as olfactory tissues, whereas legs and residual bodies were designated as non-olfactory tissues. Only 6 of the 21 putative AalbOBP genes (AalbOBP20, 59, 24, 37, 38, 39, 56) were detected in olfactory tissues while the others had some representation in non-olfactory tissues (Fig. 3). AalbOBP37 and AalbOBP39 were detected exclusively in antennae suggesting that they are probably involved in olfactory reception [12]. To confirm it, we increased PCR cycle and/or quantity of cDNA template and the results showed that AalbOBP37 and AalbOBP39 were expressed exclusively in the *Ae. albopictus* antennae (data not showed).

### Structural Predictions and Binding Affinities of AalbOBP37 and AalbOBP39

AalbOBP39 has 98% amino acid sequence identity with AaegOBP39 known as a “true” OBP [22] found in the antennae, which are associated with odorant reception. These two OBPs display a high degree of similarity in their overall structure (Fig. S3). Comparisons of AalbOBP39, AaegOBP39, CquiOBP1 and AgamOBP1 likewise showed a similar structure (Fig. S3). Similar structures likely reflect similar function [17], [28], [39], [41]–[43]. We propose that AalbOBP39 is a potential OBP involved in olfactory reception.

**Figure 4:**
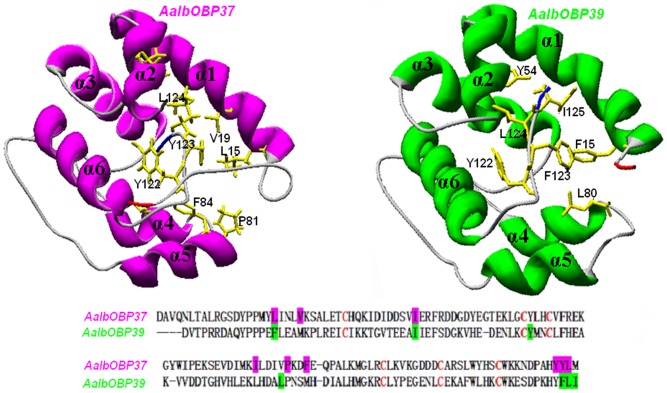
Sequences and 3D model of *Ae. albopicus* OBP37 and OBP39. The two OBPs, despite having a low amino acid sequence identity of 33%, show a remarkable similarity in their overall structures. In both cases, the hydrophobic cavity is lined with several hydrophobic amino acids, such as branched chain amino acids (Ile, Leu, Val) and aromatic residue (Tyr, Phe), which were highlighted in the sequences. For AalbOBP37, we used the CquiOBP1 (PDB: 2L2C_A) as a template. Amino acid identity between the two proteins is 34%. For AalbOBP39 the template was the structure of AaegOBP1, whose acc. No. is 3K1E_B (identity between the two proteins: 98%). N-terminus was dyed in red while C-terminus in blue.

AalbOPB37 has 92% amino acid sequence identity with AaegOBP37 and their exact functions are not clear. The overall structure of AalbOBP37 is similar to that of AalbOBP39, although they share low amino acid sequence identity (33.3%) (Fig. 4). The structural similarity supports our hypothesis that AalbOBP37 may share an overlapping function with AalbOBP39.

**Figure 5:**
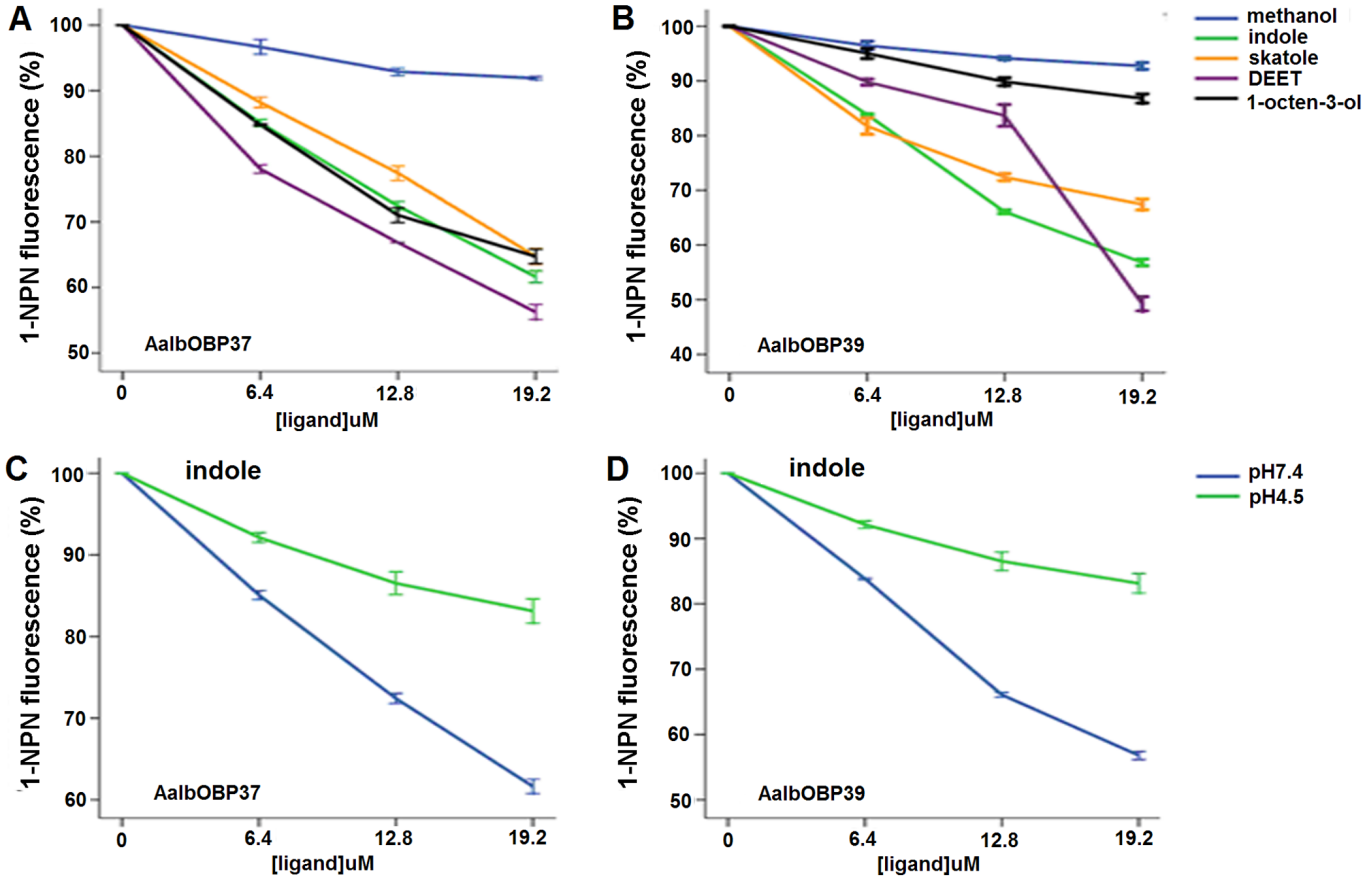
Binding affinities of AalbOBP37 and AalbOBP39. At pH 7.4, AalbOBP37 (A) and AalbOBP39 (B) bind four selected ligands, and their affinities to indole were higher at pH 7.4 than those at pH 4.5 (C&D), which showed the binding affinities of AalbOBP37 and AalbOBP39 were affected by different pH condition. Methanol was used as solvent and showed the lowest binding affinity to both OBPs. Error bars show standard deviation.

Fluorescent binding assay has been applied to study the binding affinities of OBPs to putative ligands. The fluorescent probe, 1-NPN, is most widely used in this assay [33]. It is possible to measure the affinities of a ligand by its ability to compete with 1-NPN, which is reflected as the decrease of fluorescence. As showed in Fig. 5, AalbOBP37 and AalbOBP39 exhibited relatively strong binding to both indole and skatole. However, binding affinities of AalbOBP37 to two hydrophobic molecules (DEET and 1-octen-3-ol) were different from those of AalbOBP39. Although AalbOBP37 showed high affinity to DEET and 1-octen-3-ol, AalbOBP39 showed relatively low affinity to DEET and 1-octen-3-ol, especially 1-octen-3-ol. The difference between these two OBPs might result from the number of hydrophobic amino acids in the center of the binding cavity being higher in AalbOBP37 than AalbOBP39 (Fig. 4). The spatial structure, as well as hydrophobic cavity, is expected to affect binding affinities of OBPs to ligands [28], [39].

OBPs should moreover have a property to release the ligands from their binding cavities. The low pH micro-environment plays an important role. Several studies demonstrated that the membrane surface around the dendrite of insect sensilla is negatively-charged, which induces a drop in pH in close proximity of the receptors [44], [45]. AalbOBP37 and AalbOBP39 showed high binding affinities to indole at pH 7.4, but lower at pH 4.5 (Fig. 5C&5D), which is the same as other insect OBPs. CquiOBP1 bound with MOP at high pH but not at low pH [8], [15]. AaegOBP1 has a pH-sensitive “Lid” and changed its conformation at different pH [39]. Thus, a pH-dependent manner is also likely to be a property of AalbOBP37 and AalbOBP39.

### EAG Analyses in the Control and Knock-down Mosquitoes

**Figure 6:**
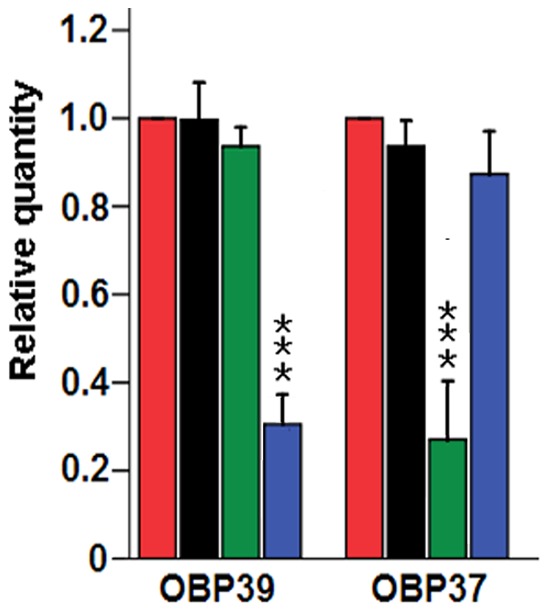
The qRT-PCR results of RNAi treatment with dsRNA of AalbOBP37 and AalbOBP39. Red column: non-injected group, as a calibrant; black column: dsRNA-dsRED injected group; green column: dsRNA-OBP37 injected group; blue column: dsRNA-OBP39 injected group. The results were analyzed using the 2^−ΔΔCT^ method, and the 2^−ΔΔCT^ value of calibrant equals to 1. Error bars represent the standard deviation of the 2^−ΔΔCT^ value. Beta-actin was used as reference gene.

Messenger RNA abundances post-treatment with dsRNA of AalbOBP37 and AalbOBP39 were measured by qRT-PCR. The housekeeping gene in *Ae. albopictus*, β-actin gene, was used to normalize the relative expressional level. The choice of this reference gene was based on previous reports on quantitative analysis of the expression of *Ae. albopictus* genes, including OBP genes [14], [34]. As described in figure 6, when injecting dsRNA-OBP37, the mRNA abundance of this gene showed a significant decrease. Additionally, while injecting dsRNA-OBP39, the OBP37 mRNA level showed no difference with dsRNA-dsRED and normal controls. The similar phenomenon was found for the AalbOBP39 treatment groups. These results support the conclusion that the decrease of mRNA abundance primarily resulted from RNAi.

**Figure 7:**
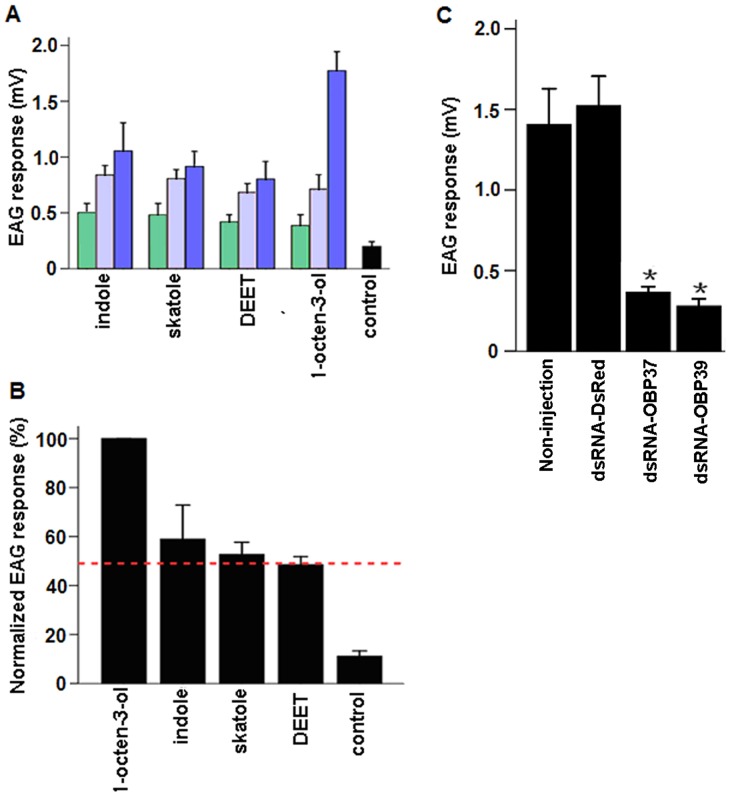
EAG responses of female *Ae. albopictus* antennae. (A) EAG response recording from female antennae showed a dose-dependent manner. Doses of selected ligands: 25 ug (green), 50 ug (purple) and 100 ug (blue), n = 6–8; mean ± std. (B) Doses of four compounds, 100 ug; n = 6–8; mean ± std, mosquito age: 4–5 days old. EAG response of 1-octen-3-ol was used as a standard (100%) to normalize those elicited by other chemicals. Indole and skatole elicited more than 50% of the 1-octen-3-ol response. (C) EAG response post-RNAi-treatment. Compound: indole, 100 ug, n = 6–8; mean±std; mosquito: 4–5 day post RNAi treatment. Detection was separated into non-injected group, dsRNA-dsRED injected group, dsRNA-OBP37 injected group and dsRNA-OBP39 injected group. Hexane (10 ul) was used as a control in this study.

To investigate the potential impact of AalbOBP37 and AalbOBP39 knockdown, we performed initial EAG screening to identify compounds that may elicit EAG response in mosquitoes prior to dsRNA treatment. We identified four selected odorants that elicited antennal responses from female *Ae. albopictus* (Fig. 7A). These four known mosquito related odorants have antennal activity in a dose-dependent manner (Fig. 7A). The responses were the highest at the 100 ug source dose. Indole, which elicited more than 50% of 1-octen-3-ol activity at 100 ug (Fig. 7B), was used to assess the function of AalbOBP37 and AalbOBP39 post RNAi-treatment. For both OBPs, EAG responses were reduced significantly in RNAi-treated mosquito antennae, compared with controls (Fig. 7C). These data support the conclusion that AalbOBP37 and AalbOBP39 are involved in *Ae. albopictus* antennal response, which is one of primary steps of olfactory reception.

### Behavioral Experiments

Mosquito behavior is modulated by the olfactory system and OBPs may act as an imperative “monitor” for the surrounding environment. Compounds that modulate behavior can have a dose-dependent effect resulting in contrasting responses. For example, the oviposition pheromone, n-heneicosane, an attractant at 1–10 ug/ml, repels *Ae. aegypti* when its source dose was higher than 100 ug [46]. *Ae. albopictus* displayed repellency when the source dose of skatole was higher than 50 ug while it is an attractant at a dose of 25 ug. The attraction weakened at a source dose of 12.5 ug (Fig. S4). The data guided our selection of 25 ug as the optimal source dose to detect the effects of other compounds.

**Figure 8:**
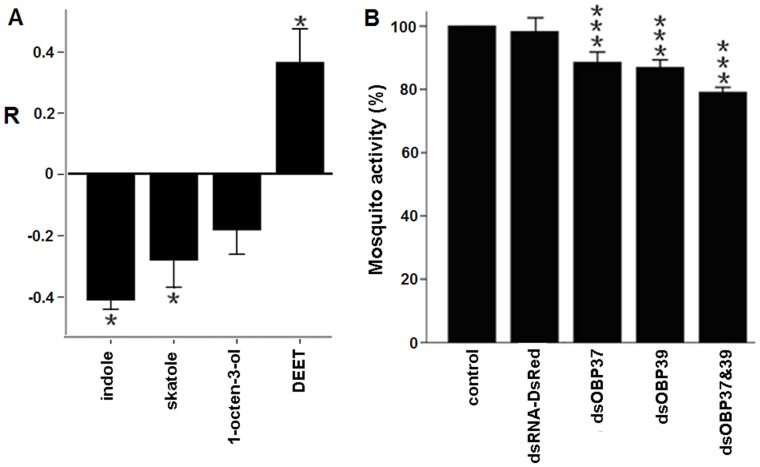
Behavior change of mosquitoes to odor compounds. (A) Repellency of EAG-activity compounds against *Ae. albopicus* female mosquitoes, which was recorded by repellency index (**R**). Compound dose: 25 ug; n = 6–8; mean ± std. Indole *X^2^* = 5.26, skatole *X^2^* = 4.17, 1-octen-3-ol *X^2^* = 2.91, DEET *X^2^* = 4.17. The repellency of each compound tested was analyzed on the basis of total number of mosquitoes in each olfactometer’s arm by the chi-square goodness-of-fit test. The test assesses whether a significant difference exists between the observed number of mosquitoes in each arm of the olfactometer and the expected number based upon the null hypothesis of a 50∶50 mosquito’s distribution. (B) Chemotaxis activities of mosquito in different treatment groups were detected: Control (non-injected), negative control (dsRNA-dsRED injected), dsRNA-OBP37 injected group; dsRNA-OBP39 injected group and dsRNA-OBP37&OBP39 injected group. Indole dose: 12.5 ug in each arm; n = 6–8; mean±std. The data showed significant difference compared with controls (P<0.001).

Indole, skatole and 1-octen-3-ol showed attraction and DEET displayed repellency as expected (Fig. 8A). Indole elicited greater attraction behavior than other compounds. RNAi-mediated suppression of AalbOBP37 or AalbOBP39 modulated the activity of mosquitoes ∼10% in single treated insects (P<0.001), and ∼20% when both target genes were knocked down simultaneously (p<0.001) (Fig. 8B). These results provided strong evidence that AalbOBP37 and AalbOBP39 were involved in *Ae.albopictus* olfactory reception. However, while RNAi-mediated knockdown resulted in an approximate 70% reduction of mRNA abundance (Fig. 6), olfactory activity of double knock-down mosquitoes (OBP37 and OBP39 together) was still high, ∼80% (Fig. 8B). These data support the hypothesis that other OBP genes expressed in antennae likely may have a role in olfactory reception. The genome of *Ae. albopictus* is being sequenced. Further molecular and functional analysis of other OBPs will improve our understanding of olfaction of this invasive vector species and contribute to the development of more efficient and environmentally-friendly mosquito control strategies.

## Conclusion

This work presents for the first time a large-scale cloning and identification of genes encoding OBPs from *Ae. albopictus*. Functional analysis, EAG response and behavioral experiments, demonstrated these two antenna-specific OBPs were involved in olfactory reception.

## Supporting Information

Figure S1 **Binding curves of AalbOBP37 and AalbOBP39 to 1-NPN.** A. 25 ug/ml of AalbOBP37 at pH 7.4, 20 ul of 1-NPN (3.2 mM in methanol) was needed to saturate the fluorescence intensity while 25 ug/ml of AalbOBP39 was saturated with 19 ul of 1-NPN, the final concentration of which in protein solution was 32 uM and 30.4 uM, respectively. B. Binding affinities of OBPs and *E.coli* protein to 1-NPN. The target OBPs could bind 1-NPN, which showed increasing fluorescent intensity, comparing with negative control. The proteins of *E.coli* showed no binding affinity to 1-NPN. The fluorescent of *E.coli* protein may derive from the intrinsic fluorescence of tryptophan residues. Protein concentration: 25 ug/ml in 20 mM sodium acetate solution. Ligand: 1-NPN in methanol solution. Purple spot showed the fluorescent of sodium acetate solution.
(TIF)

Figure S2 **Amino acid alignment of** ***Ae. albopictus*** **Plus-C OBPs. A.** The Plus-C OBPs showed six conserved cysteine residues, as well as two additional conserved cysteine residues (C4a and C6a) in red and one conserved proline residue in blue. Asterisk shows the conserved residues. **B.** Phylogenic analysis separated 21 putative AalbOBPs into two groups, classic OBP and Plus-C OBP.
(TIF)

Figure S3 **Structural models of AalbOBP39, AaegOBP1, CquiOBP1 and AgamOBP1.** The OBPs have high amino acid sequence identity (92%) and show similarity in their overall structures. Identical amino acids are colored in gray.
(TIF)

Figure S4 **Repellency of skatole to** ***Ae. albopictus*** **mosquitoes in different concentrations.** The repellency of each concentration was analyzed on the basis of total number of mosquitoes in each olfactometer’s arm by the chi-square goodness-of-fit test. The test assesses whether a significant difference exists between the observed number of mosquitoes in each arm of the olfactometer and the expected number based upon the null hypothesis of a 50∶50 mosquito’s distribution. The *X^2^* value of each concentration: 12.5 ug *X^2^* = 0.167, 25 ug *X^2^* = 5.26, 50 ug *X^2^* = 1.19, 100 ug *X^2^* = 4.84.
(TIF)

Table S1 **List of primers designed for cloning 5′ RACE sequences of AalbOBP cDNA sequences.**
(DOCX)

Table S2 **List of primers designed for cloning 3′ RACE sequences of AalbOBP cDNA sequences.**
(DOCX)

Table S3 **List of gene specific primers used in RT-PCR experiments.**
(DOCX)

Table S4 **List of primers utilized for vector construction, dsRNA synthesis and qRT-PCR studies.**
(DOCX)

Table S5 **Homology of** ***Ae. albopictus*** **with other mosquito OBPs.**
(DOCX)
