# Supplementary figures and images for: Molecular and Functional Characterization of Odorant-Binding Protein Genes in an Invasive Vector Mosquito, Aedes albopictus
Source: PLoS One. 2013 Jul 23;8(7):e68836. doi: 10.1371/journal.pone.0068836 (PMC3720860; doi:10.1371/journal.pone.0068836)

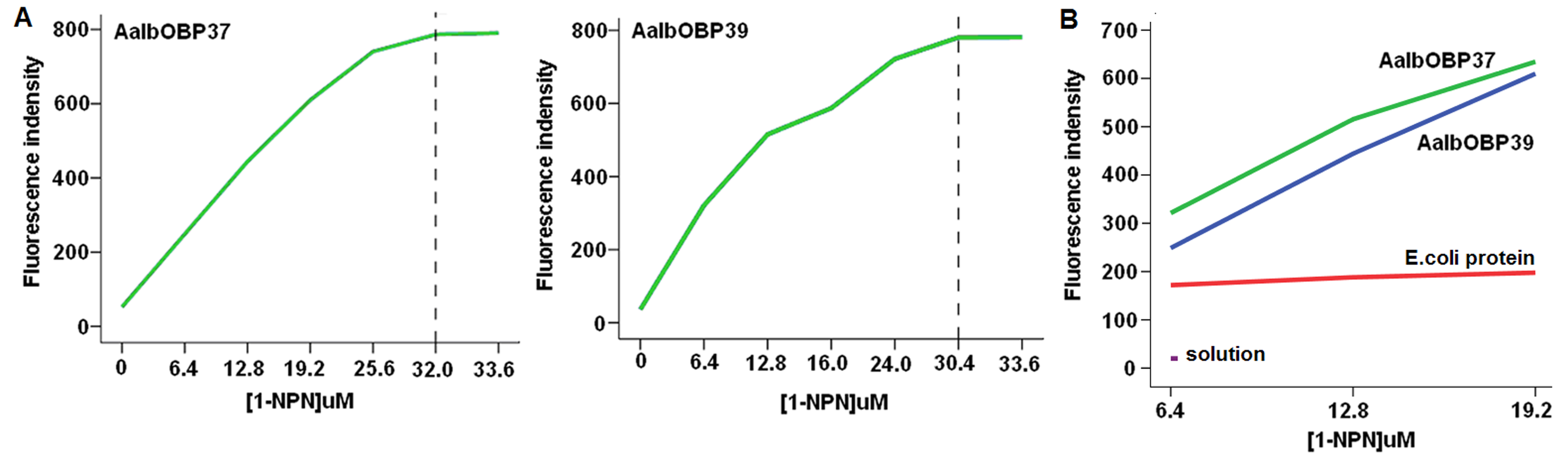

Supplement: Figure S1 — Binding curves of AalbOBP37 and AalbOBP39 to 1-NPN. A. 25 ug/ml of AalbOBP37 at pH 7.4, 20 ul of 1-NPN (3.2 mM in methanol) was needed to saturate the fluorescence intensity while 25 ug/ml of AalbOBP39 was saturated with 19 ul of 1-NPN, the final concentration of which in protein solution was 32 uM and 30.4 uM, respectively. B. Binding affinities of OBPs and E.coli protein to 1-NPN. The target OBPs could bind 1-NPN, which showed increasing fluorescent intensity, comparing with negative control. The proteins of E.coli showed no binding affinity to 1-NPN. The fluorescent of E.coli protein may derive from the intrinsic fluorescence of tryptophan residues. Protein concentration: 25 ug/ml in 20 mM sodium acetate solution. Ligand: 1-NPN in methanol solution. Purple spot showed the fluorescent of sodium acetate solution. (TIF) [file pone.0068836.s001.tif]

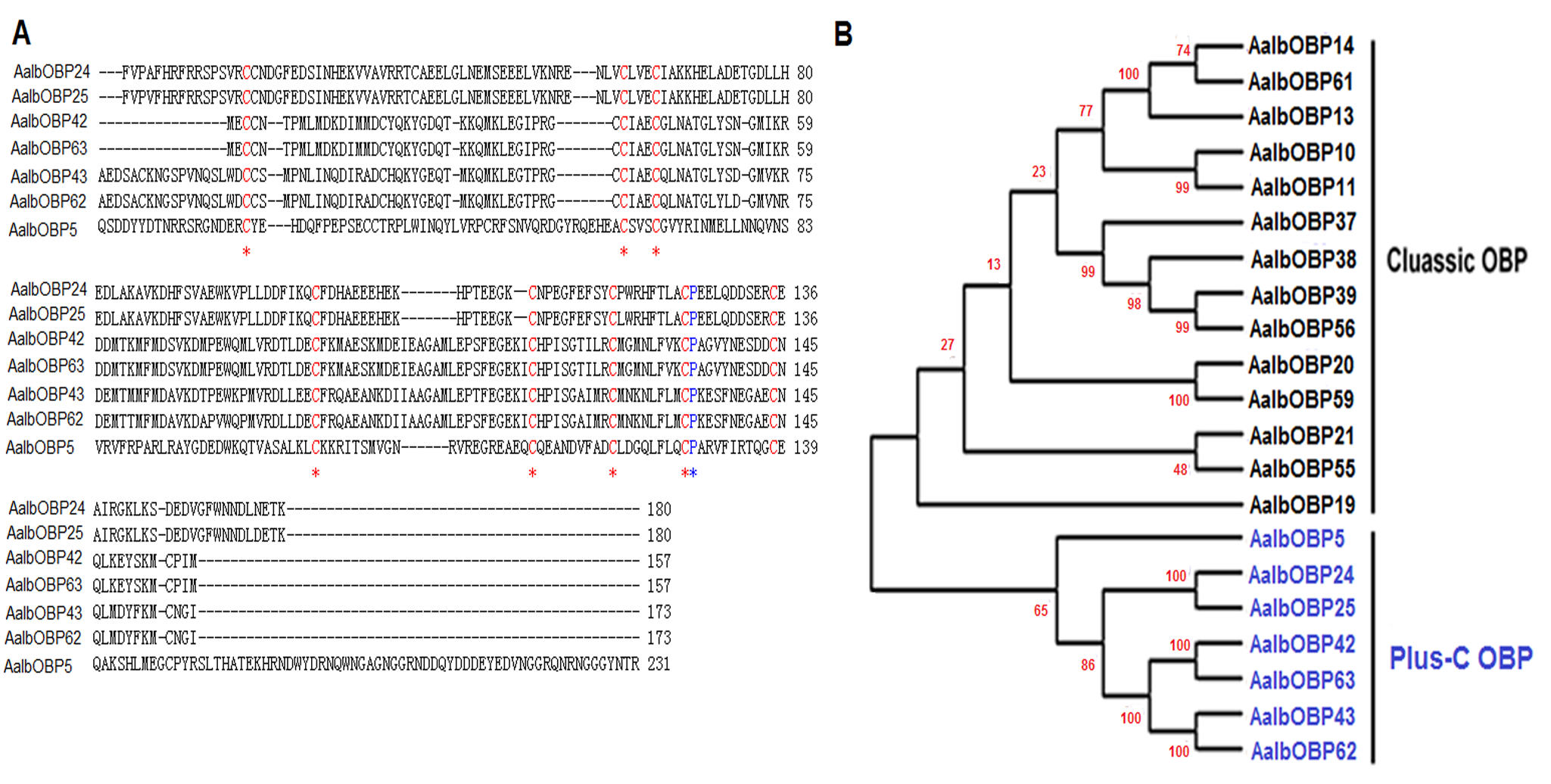

Supplement: Figure S2 — Amino acid alignment of Ae. albopictus Plus-C OBPs. A. The Plus-C OBPs showed six conserved cysteine residues, as well as two additional conserved cysteine residues (C4a and C6a) in red and one conserved proline residue in blue. Asterisk shows the conserved residues. B. Phylogenic analysis separated 21 putative AalbOBPs into two groups, classic OBP and Plus-C OBP. (TIF) [file pone.0068836.s002.tif]

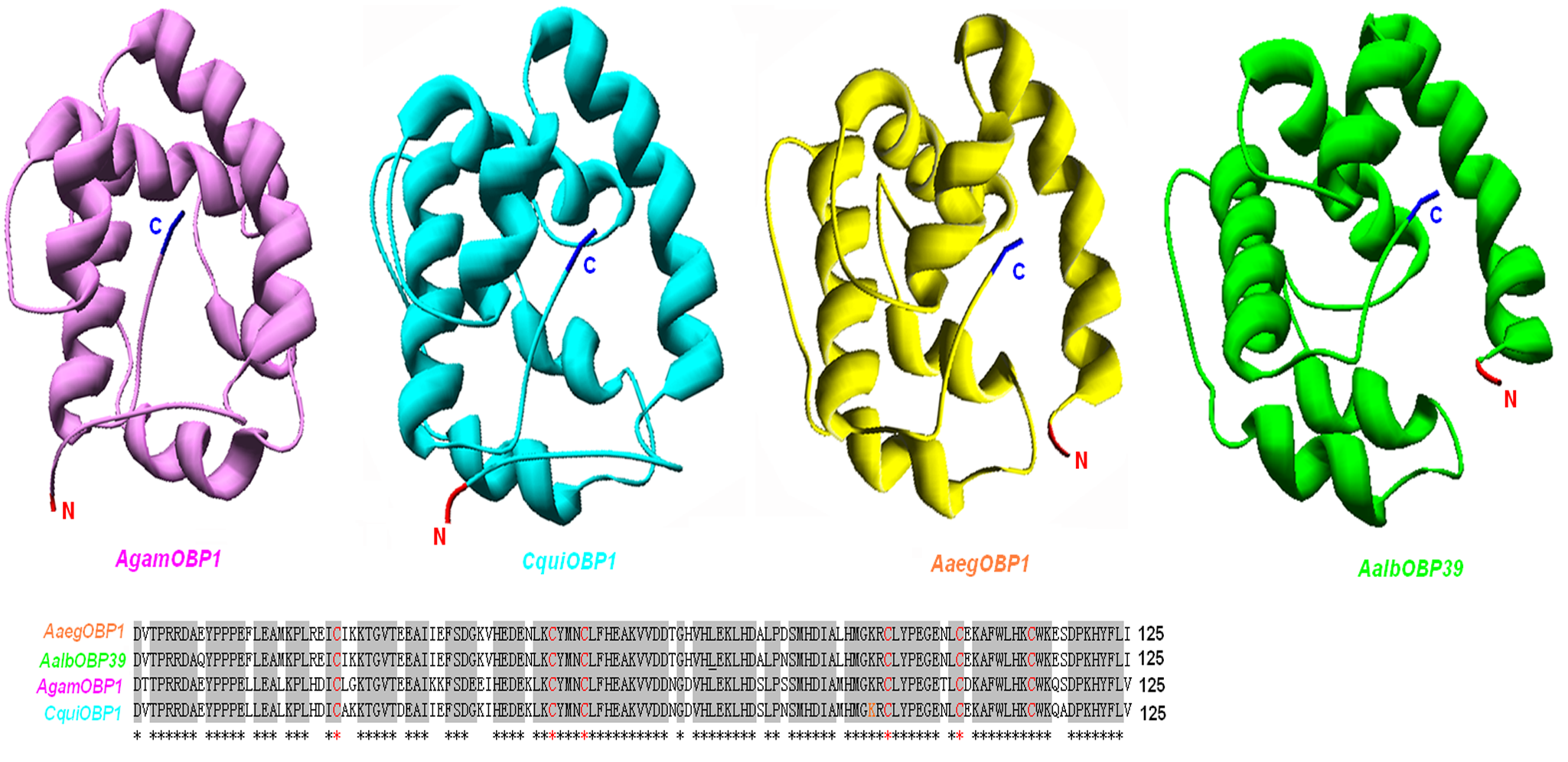

Supplement: Figure S3 — Structural models of AalbOBP39, AaegOBP1, CquiOBP1 and AgamOBP1. The OBPs have high amino acid sequence identity (92%) and show similarity in their overall structures. Identical amino acids are colored in gray. (TIF) [file pone.0068836.s003.tif]
