# Supplementary material for: Molecular and Functional Characterization of Odorant-Binding Protein Genes in an Invasive Vector Mosquito, Aedes albopictus
Source: PLoS One. 2013 Jul 23;8(7):e68836. doi: 10.1371/journal.pone.0068836 (PMC3720860; doi:10.1371/journal.pone.0068836)
Supplement: Table S1 — List of primers designed for cloning 5′ RACE sequences of AalbOBP cDNA sequences. (DOCX) [file pone.0068836.s005.docx]

**Table S1. List of primers designed for cloning 5’-RACE sequences of AalbOBP cDNA sequences**

| OBP Names | Outer primers | inner primers |
| --- | --- | --- |
| AalbOBP5 | CGCCTCTCGACCCTCTCTAACGCGA | CCATCCAGACAGTCGGCGAAGACGT |
| AalbOBP10 | CGTAGCACTTGATCGACTGATCGGG | TCGTTCATCAGGCGCAGCTTCGAAA |
| AalbOBP11 | CAGCTGCGTCACGATTGCTTCCTCA | TTCCTGCTGCATCCATGAAACCGGT |
| AalbOBP13 | GTTGCATGCATTTTCCGGCCAGTTG | TCTGTTCGTCGGTGACGGCAGCCAT |
| AalbOBP14 | GTTGCATGCATTTTCCGGCCAGTTG | TCTGTTCGTCGGTGACGGCAGCCAT |
| AalbOBP19 | CAGCAGCTCCATGTTCACCCCCGTC | AAACACACCGTATGAAGCAAGCCGC |
| AalbOBP20 | TCCGGTGTCGTTCAGTTCGTTCAG | CTCGACGCTGTAATCCTTCATGCT |
| AalbOBP21 | TGATGCACTTGATGACACACTTGG | CCGAATTCTTCCTGTAGATCATCC |
| AalbOBP24 | TGGAATCCTCGAATCCATCGTTGCA | CCGAAGGACTACGACGGAAGCGATG |
| AalbOBP25 | TGCATTTGCCCTCCTCGGTGGGGTG | CTCTTCGGCATGATCGAAGCATTGC |
| AalbOBP37 | ATCCCGGAATCGTTCGATGACTG | TTATCAAATACATAGGAGGGTAG |
| AalbOBP38 | AGGCTTTCTCACACAAATTGTCTCC | GAACCAGTTGAGCGTGATCAGATGC |
| AalbOBP39 | TTGATGCAAATTTCACGGAGAGG | GGTGGTGGATATTCAGCGTCTCT |
| AalbOBP42 | GGGTGGCAAATCTTCTCCCCTTCAA | CTCCAACATGGCACCGGCTTCGATT |
| AalbOBP43 | TGGCGTTCAGTTGACATTCGG | CAGCATCCACGGGGTGTTCCTTC |
| AalbOBP55 | TTGTTTGTCGCAACGCATTGAG | TTTGATTCCGTCGGAAGCGGTG |
| AalbOBP56 | CGCGCATTGAGTCGGGAAGGGCGTC | TCCAGGTGTACGTGGCCGGTATCGT |
| AalbOBP59 | TCCGGTGTCGTTCAGTTCGTTCAG | CTCGACGCTGTAATCCTTCATGCT |
| AalbOBP61 | GTTGCATGCATTTTCCGGCCAGTTG | TCTGTTCGTCGGTGACGGCAGCCAT |
| AalbOBP62 | TGGCGTTCAGTTGACATTCGG | CAGCATCCACGGGGTGTTCCTTC |
| AalbOBP63 | TCTTCTCCCCCTCGAAACTCGGCTC | GGACTCGGCCATTTTGAAGCACTCG |

5’-Full RACE Core Set kit (Takara) and SARMT RACE cDNA Amplification kit (Clontech) were used. Specific primers were designed according to the instructions of the manufactures.

| Outer primers (Mix) |  |
| --- | --- |
| CTAATACGACTCACTATAGGGCAAGCAGTGGTATCAACGCAGAGT | Long(0.4uM) |
| CTAATACGACTCACTATAGGGC | Short(2uM) |
| inner primers |  |
| AAGCAGTGGTATCAACGCAGAGT | (10uM) |

The 5’ RACE universal primers of SARMT RACE cDNA Amplification kit (Clontech) were used in the manuscript.

| Outer primers (Mix) |  |
| --- | --- |
| CATGGCTACATGCTGACAGCCTA | (10uM) |
| inner primers |  |
| CGCGGATCCACAGCCTACTGATGATCAGTCGATG | (10uM) |

The 5’ RACE universal primers of 5’-Full RACE Core Set kit (Takara) were used in the manuscript.
