# Supplementary material for: Molecular and Functional Characterization of Odorant-Binding Protein Genes in an Invasive Vector Mosquito, Aedes albopictus
Source: PLoS One. 2013 Jul 23;8(7):e68836. doi: 10.1371/journal.pone.0068836 (PMC3720860; doi:10.1371/journal.pone.0068836)
Supplement: Table S2 — List of primers designed for cloning 3′ RACE sequences of AalbOBP cDNA sequences. (DOCX) [file pone.0068836.s006.docx]

**Table S2. List of primers designed for cloning 3’-RACE sequences of AalbOBP cDNA sequences**

| OBP names | Outer primers | inner primers |
| --- | --- | --- |
| AalbOBP5 | ACGTCTTCGCCGACTGTCTGGATGG | CAACTGTTCCTGCAATGCCCCGCAC |
| AalbOBP10 | CATAGCCAATCCCGATCAGTCGATC | CCAGTGCTTCTTTTCGAAGCTGCGC |
| AalbOBP11 | ACCGGTTTCATGGATGCAGCAGGAA | GGAAGCAATCGTGACGCAGCTGAGC |
| AalbOBP13 | CGGAAAGTCACGATGCGGCTTGCTT | GGAGCGTATGATGATCAGACGGGGG |
| AalbOBP14 | CGGAAAGTCACGATGCGGCTTGCTT | GGAGCGTATGATGATCAGACGGGGG |
| AalbOBP19 | AAGGATTGTTTGGCGCTGCGGGACG | GGTCGAGTGCTACTGCATGAAGGCG |
| AalbOBP20 | CGGGCGTATTTTCTAATGCGGTGTG | TGGTGGAAGGCAGGAGCAAAGACAG |
| AalbOBP21 | TGGATCCCGAGGATGTTTGCGAGCG | GCCTATGCGCTGTATCAGTGCATCC |
| AalbOBP24 | CACGAAAAGGTAGTCGCCGTGAGAC | CTGCGCCGAAGAGCTGGGACTGAAC |
| AalbOBP25 | CCGTGAAAATCTGGTTTGCCTGGTG | GCCAAAAAGCACGAACTGGCAGACG |
| AalbOBP37 | GATTCAGTCATCGAACGATTCCGGG | CGAAGGAACTGAGAAGCTGGGATGC |
| AalbOBP38 | ATGCATCTGATCACGCTCAACTGG | CAATGCATCTGATCACGCTCAACTG |
| AalbOBP39 | ATCCACCACCCGAGTTCTTGGAGGC | GGCAATCATTGAATTCAGCGATGGC |
| AalbOBP42 | CATGGATTCGGTCAAGGACATGCCC | ATGCTCGTCCGCGATACGTTGGACG |
| AalbOBP43 | TTGATCAACCAGGACATCCGTGCGG | AGAAGGAACACCCCGTGGATGCTGC |
| AalbOBP55 | CTGAATGCATGTCGCACCGCTTCC | CTGCGATGCATCGTACGCCATTGCT |
| AalbOBP56 | GATACCGGCCACGTACACCTGGAGA | ACACGACGCCCTTCCCGACTCAATG |
| AalbOBP59 | CGGGCGTATTTTCTAATGCGGTGTG | TGGTGGAAGGCAGGAGCAAAGACAG |
| AalbOBP61 | CGGAAAGTCACGATGCGGCTTGCTT | GGAGCGTATGATGATCAGACGGGGG |
| AalbOBP62 | CCGGGACGAGATGACGACGATGTT | TGCGGTCAAGGATGCACCCGTATGG |
| AalbOBP63 | TTCCGTCAAGGACATGCCCGAGTGG | CCGCGATACGTTGGACGAGTGCTTC |

SMART RACE cDNA Amplification kit (Clontech) was used. Specific primers were

designed according to the instructions of the manufactures.

| Outer primers (Mix) |  |
| --- | --- |
| CTAATACGACTCACTATAGGGCAAGCAGTGGTATCAACGCAGAGT | Long(0.4uM) |
| CTAATACGACTCACTATAGGGC | Short(2uM) |
| inner primers |  |
| AAGCAGTGGTATCAACGCAGAGT | (10uM) |

The 3’ RACE universal primers of SARMT RACE cDNA Amplification kit (Clontech) were used in the manuscript.
