# Supplementary material for: Molecular and Functional Characterization of Odorant-Binding Protein Genes in an Invasive Vector Mosquito, Aedes albopictus
Source: PLoS One. 2013 Jul 23;8(7):e68836. doi: 10.1371/journal.pone.0068836 (PMC3720860; doi:10.1371/journal.pone.0068836)
Supplement: Table S3 — List of gene specific primers used in RT-PCR experiments. (DOCX) [file pone.0068836.s007.docx]

**Table S3. List of gene specific primers used in RT-PCR experiments**

| OBP names | Primer Forward 5’-3’ | Primer Reverse 5’-3’ |
| --- | --- | --- |
| AalbOBP5 | ATGGTTTCCAAACTGTGGATACTCTTGG | TTGACCATTGTTTCTATTTCTATCG |
| AalbOBP10 | ATGACATCATTCCGAATGGTGAATG | TCAGAGCACCAGCTTGGCCTTGTT |
| AalbOBP11 | ATGAGACTTTCGGTTTTCATTGCGT | CTAAAACAATCCAGCTTTGTTTTTG |
| AalbOBP13 | GTGGCCATTATTTCGCTTGCCC | TCACATCAAACTAGCACGGTTGGC |
| AalbOBP14 | ACCTTTGTGGCCATTATTTCGCTTG | ACATCAAACTAGCACGGTTTGCAACA |
| AalbOBP19 | ATGACTTCAAACCATTGTTGGATCG | TCACAAGACATTCCGCTTCTTGAAC |
| AalbOBP20 | GAAACCTCTCACAAAGTTATCTCTGCC | GCTCCTGCCTTCCACCACATGG |
| AalbOBP21 | GAGTGGATCATCGTGTTAACGAG | CAGCGCATAGGCCCGCTCGC |
| AalbOBP24 | CCATCAATCACGAAAAGGTAGTCG | CTCGCACCGTTCCGAATCATC |
| AalbOBP25 | CCATCAACCACGAGAAGGTGGTC | CTTCGCACCGTTCGGAATCGTC |
| AalbOBP37 | GCTACTGTTTCTGAGTCATGGGGATGC | AGTAACCCTTTTCGCGAAAAACG |
| AalbOBP38 | ACTGCCAAGAACCAAGACGAGATGC | TAGTGCACTGGGTCTCGCTCTTTCC |
| AalbOBP39 | GTAGCTTTGGTTATAAGTGC | CGCAAAGGTTTTCTCCCTCTG |
| AalbOBP42 | TGGAGGGCATCCCTCGKGG | CGGRTGRCAAATCTTCTCCCCYTCA |
| AalbOBP43 | GCTTGTGGGATTGTTGCAGTATGCC | GAAGCATTCSTCSAGCAGATCACGT |
| AalbOBP55 | GACCAAACGAGTGGATCTTGTGC | CGGATTGTTTGTCGCAACGC |
| AalbOBP56 | GCAAGTTGTGTAGTTGCATTGTCGG | CCTTCCAGCACTTGTGCAACC |
| AalbOBP59 | GAAGCCTCTTACAAAGTTATCTCTGCC | GCTCCTGCCTTCCACCAAATGG |
| AalbOBP61 | ATGNAAACCTTTGTGGCCATTATTTCGC | TACATCAAACTAGCACGGTTTGCAAC |
| AalbOBP62 | GCTTGTGGGATTGTTGCAGTATGCC | ATTCSTCSAGCAGGTCGCGAACCAT |
| AalbOBP63 | CTCGKGGTTGTTGCATTGCCG | GGRTGRCAAATCTTCTCCCCYTCGA |
