# Supplementary material for: Molecular and Functional Characterization of Odorant-Binding Protein Genes in an Invasive Vector Mosquito, Aedes albopictus
Source: PLoS One. 2013 Jul 23;8(7):e68836. doi: 10.1371/journal.pone.0068836 (PMC3720860; doi:10.1371/journal.pone.0068836)
Supplement: Table S4 — List of primers utilized for vector construction, dsRNA synthesis and qRT-PCR studies. (DOCX) [file pone.0068836.s008.docx]

**A Primers for insert amplification to construct the expression vector**

| Primer names | Primer sequences |
| --- | --- |
| fw AalbOBP37-MscI | 5’-GGG**TGG/CCA**CAGTTCAAAATCTAACCGC-3’ |
| rv AalbOBP37-HindIII | 5’- CCC**A/AGCTT**CATTAAGTAATAGTGC-3’ |
| fw AalbOBP39-MscI | 5’-GGG**TGG/CCA**GCGATGTAACTCCTCGCAG-3’ |
| rv AalbOBP37-HindIII | 5’-CCC**A/AGCTT**GATCAGGAAGTAATGC-3’ |

The specific primers were used for construction of expression vector, enzyme restriction sites were underlined.

**B Primers for dsRNA synthesis**

| Primer names | Primer sequences |
| --- | --- |
| fw-AalbOBP37-T7 | **TAATACGACTCACTATAGGG**GTGTCGTAAGCTACTTTGGGTG |
| rv-AalbOBP37 | CCACAAAGACCTAGCGCAATCATC |
| fw-AalbOBP37 | GTGTCGTAAGCTACTTTGGGTGTG |
| rv-AalbOBP37-T7 | **TAATACGACTCACTATAGGG**CCACAAAGACCTAGCGCAATC |
| fw-AalbOBP39-T7 | **TAATACGACTCACTATAGGG**ACAGTATCCACCACCCGA |
| rv-AalbOBP39 | GATCAGGAAGTAATGCTTTG |
| fw-AalbOBP39 | ACAGTATCCACCACCCGA |
| rv-AalbOBP39-T7 | **TAATACGACTCACTATAGGG**GATCAGGAAGTAATGCTTTG |
| fw-dsRed-T7 | **TAATACGACTCACTATAGGG**GCTCCTCCAAGAACGTC |
| rv- dsRed | GCGCTCGTACTGCTCCAC |
| fw-dsRed | GCTCCTCCAAGAACGTC |
| rv- dsRed -T7 | **TAATACGACTCACTATAGGG**GCGCTCGTACTGCTCCAC |

The primers with T7 promoter were used to synthesize dsRNA in vitro. AalbOBP37 and AalbOBP39 were target genes, while the dsRed was used as negative control. T7 promoter was underlined.

**C Primers for qRT-PCR**

| Primer names | Primer sequences |
| --- | --- |
| AalbOBP37-F | GAACTGAGAAGCTGGGATG |
| AalbOBP37-R | GTGCTGGTTGCTCAAAGTC |
| AalbOBP39-F | TTCAGCGATGGCAAAGTCC |
| AalbOBP39-R | TGCCCAGTATCGTCAACAACC |
| β-actin-F | GCCGTCTTCCCGTCCAT |
| β-actin-R | GGCGACACGCAGCTCATT |

The primers of AalbOBP37 and AalbOBP39 for qRT-PCR generated a product of 128bp and 92bp, respectively; while primers of β-actin generated a product of 206bp.
