# Supplementary material for: Molecular and Functional Characterization of Odorant-Binding Protein Genes in an Invasive Vector Mosquito, Aedes albopictus
Source: PLoS One. 2013 Jul 23;8(7):e68836. doi: 10.1371/journal.pone.0068836 (PMC3720860; doi:10.1371/journal.pone.0068836)
Supplement: Table S5 — Homology of Ae. albopictus with other mosquito OBPs. (DOCX) [file pone.0068836.s009.docx]

**Table S5. Homology of *Ae. albopictus* with other mosquito OBPs.**

| OBP name | Phylogenetic Group | *Ae.aegypt* homolog | Protein identity | *Cx.quinquefasciatus* homolog | Protein identity | *An.gambiae* homolog | Protein identity |
| --- | --- | --- | --- | --- | --- | --- | --- |
| AalbOBP5 | Plus-C | AaegOBP5 | 84% |  |  |  |  |
| AalbOBP10 | GroupB | AaegOBP10 | 86% | CquiOBP24 | 65% | AgamOBP10 | 44% |
| AalbOBP11 | GroupB | AaegOBP11 | 91% | CquiOBP19 | 67% | AgamOBP25 | 50% |
| AalbOBP13 | GroupB | AaegOBP61 | 93% | CquiOBP17 | 73% | AgamOBP67 | 68% |
| AalbOBP14 | GroupB | AaegOBP14 | 92% | CquiOBP17 | 73% | AgamOBP67 | 68% |
| AalbOBP19 | GroupB | AaegOBP19 | 82% | CquiOBP52 | 34% |  |  |
| AalbOBP20 | PBPRP4 | AaegOBP20 | 91% | CquiOBP14 | 74% | AgamOBP66 | 54% |
| AalbOBP21 | GroupB | AaegOBP21 | 89% | CquiOBP43 | 34% | AgamOBP9 | 38% |
| AalbOBP24 | Plus-C | AaegOBP24 | 95% |  |  |  |  |
| AalbOBP25 | Plus-C | AaegOBP25 | 96% |  |  |  |  |
| AalbOBP37 | OS-E/OS-F | AaegOBP37 | 92% | CquiOBP4 | 56% |  |  |
| AalbOBP38 | OS-E/OS-F | AaegOBP38 | 98% | CquiOBP2 | 93% | AgamOBP3 | 92% |
| AalbOBP39 | OS-E/OS-F | AaegOBP39 | 98% | CquiOBP1 | 87% | AgamOBP1 | 82% |
| AalbOBP42 | Plus-C | AaegOBP42 | 97% |  |  |  |  |
| AalbOBP43 | Plus-C | AaegOBP43 | 90% |  |  |  |  |
| AalbOBP55 | OBP19a | AaegOBP55 | 92% | CquiOBP11 | 68% | AgamOBP20 | 63% |
| AalbOBP56 | OS-E/OS-F | AaegOBP56 | 95% | CquiOBP1 | 88% | AgamOBP1 | 82% |
| AalbOBP59 | PBPRP4 | AaegOBP59 | 90% | CquiOBP14 | 72% | AgamOBP66 | 54% |
| AalbOBP61 | GroupB | AaegOBP61 | 93% | CquiOBP17 | 73% | AgamOBP67 | 68% |
| AalbOBP62 | Plus-C | AaegOBP62 | 90% |  |  |  |  |
| AalbOBP63 | Plus-C | AaegOBP63 | 97% |  |  |  |  |
